# Orion: Detecting regions of the human non-coding genome that are intolerant to variation using population genetics

**DOI:** 10.1371/journal.pone.0181604

**Published:** 2017-08-10

**Authors:** Ayal B. Gussow, Brett R. Copeland, Ryan S. Dhindsa, Quanli Wang, Slavé Petrovski, William H. Majoros, Andrew S. Allen, David B. Goldstein

**Affiliations:** 1 Program in Computational Biology and Bioinformatics, Duke University, Durham, NC, United States of America; 2 Institute for Genomic Medicine, Columbia University, New York, NY, United States of America; 3 Department of Medicine, The University of Melbourne, Austin Health and Royal Melbourne Hospital, Melbourne, Victoria, Australia; 4 Center for Genomic and Computational Biology, Duke University, Durham, NC, United States of America; 5 Department of Biostatistics and Bioinformatics, Duke University, Durham NC, United States of America; Case Western Reserve University School of Medicine, UNITED STATES

## Abstract

There is broad agreement that genetic mutations occurring outside of the protein-coding regions play a key role in human disease. Despite this consensus, we are not yet capable of discerning which portions of non-coding sequence are important in the context of human disease. Here, we present Orion, an approach that detects regions of the non-coding genome that are depleted of variation, suggesting that the regions are *intolerant* of mutations and subject to purifying selection in the human lineage. We show that Orion is highly correlated with known intolerant regions as well as regions that harbor putatively pathogenic variation. This approach provides a mechanism to identify pathogenic variation in the human non-coding genome and will have immediate utility in the diagnostic interpretation of patient genomes and in large case control studies using whole-genome sequences.

## Introduction

The rising prevalence of whole-genome sequencing (WGS) has led to an abundance of sequence data. The utility of WGS data in a clinical setting lies in the ability to prioritize [1] the mutations detected in patient cohorts in order to identify disease-causal mutations. We have previously introduced three population genetics-based methodologies [2–4] that can identify genomic regions in which variation is strongly selected against and are thus more likely to be pathogenic when mutated.

However, all of these methodologies are directly tied to known protein-coding genes, leaving the entire non-coding genome—which is known to carry disease-causing mutations [5,6]—untouched. Though there are many existing methods that assess the non-coding genome, these methods tend to rely heavily on conservation or functional annotations. Both of these approaches have limitations. Conservation cannot directly assess regions that have been under selection recently in the human lineage, or were under selection in the mammalian phylogeny but have lost their functionality in humans. Functional annotations can indicate the biochemical actions of a genomic region, but they cannot assess the region's likelihood of causing disease when mutated. In consequence whole genome sequence data is currently considered almost uninterpretable.

Here, we describe an approach termed Orion, which scans the entire genome for regions that are depleted of variation in the human population in comparison to expectation. Such depleted regions are considered intolerant. The Orion methodology quantifies the intolerance of a given stretch of sequence by estimating the difference between the observed and expected site-frequency spectrums (SFS). We applied this methodology to a set (n = 1,662) of WGS samples as a sliding window across the genome, calculating a regional intolerance score for each window. Each window's score was then applied to the base at the center of the window.

We assessed the Orion scores by evaluating how they behave in in comparison with a number of genomic features, including protein coding exons (known to be intolerant relative to the genome as a whole), ultra-conserved non-coding elements (UCNEs), and DNase Hypersensitive sites (DHSs). We found enrichment for intolerant Orion scores in each of the regions corresponding to these features, indicating that Orion scores do capture signals of intolerance to variation. We then used the Orion scores to differentiate the human genome into regions that are and are not intolerant. Using these demarcations, we show that intolerant regions are enriched for previously reported *de novo* mutations in patients with presumed genetic diseases and with previously reported non-coding pathogenic variants.

## Results

### Developing the Orion approach

The underlying methodology for the Orion approach is based on the difference between the expected and observed site-frequency spectrum (SFS) of a given stretch of sequence. Here, the SFS is defined as a vector in which the *i*^th^ element is equal to the number of variants in the assessed population sample that appear *i* times within the sample. Thus, the element of the SFS at *i* = 1 is equal to the number of variants that are singletons in the sample for a given window[7]. The element at *i* = 2 is equal to the number of variants that are doubletons, and so forth.

We used a WGS cohort that combined an internal cohort of unrelated controls (n = 624, S1 Table) with the unrelated parents of the Simons Foundation’s Simons Simplex Collection (n = 1,038) to calculate the Orion scores. For a region of interest, we calculated the observed SFS across this WGS control cohort (n = 1,662) and the expected SFS for the cohort under neutrality [7]. For the observed SFS, we filtered for genotype quality and coverage (Methods). The expected SFS is based on the cohort sample size, the region's mutation rate [8] and length, and the effective human population size (Methods).

We then calculated the difference between the observed and expected SFS in order to generate a score. As there are many ways to calculate the difference between two distributions, we used a forward-simulation framework [9] (Methods) to simulate different selection pressures on human populations. We then tested a number of score formulations and selected the one most correlated with selection pressures (S1 Text). Based on these evaluations we chose to use the weighted mean difference between points on the SFS, divided by the expected number of mutations introduced into the population per generation (θ). The weights for the weighted mean are derived from the inverse of the minor allele frequency (Methods), so that rare variants contribute more information to the final score. This is based on previous observations that the frequency of rare variation is highly indicative of intolerance [4].

In this formulation, expected is subtracted from observed. A higher score indicates a more intolerant region, while a lower score indicates a more tolerant one.

Note that the expected SFS in this formulation is calculated based on neutral theory, though in practice the assumptions of neutrality do not hold. As such, we do not use the absolute value of the deviation from neutrality to assess intolerance. Rather, we compare the magnitude of deviation from neutrality between regions. Throughout this article we therefore use the Orion scores in one of two ways: either by comparing the relative difference between sets of scores, or by detecting stretches of scores that empirically match known regions that are highly intolerant.

### Assessing the Orion approach on genes

We applied the Orion methodology to the exons of 1000 randomly selected genes and 1000 random stretches of non-coding sequence matched in size to the selected genes that do not overlap with repeat regions (S1 Data File) in order to assess our approach, with the expectation that protein encoding exons of genes should have higher Orion scores overall. For the genic definitions, we used the Consensus Coding DNA Sequence regions (CCDS release 14) [10].

**Fig 1:**
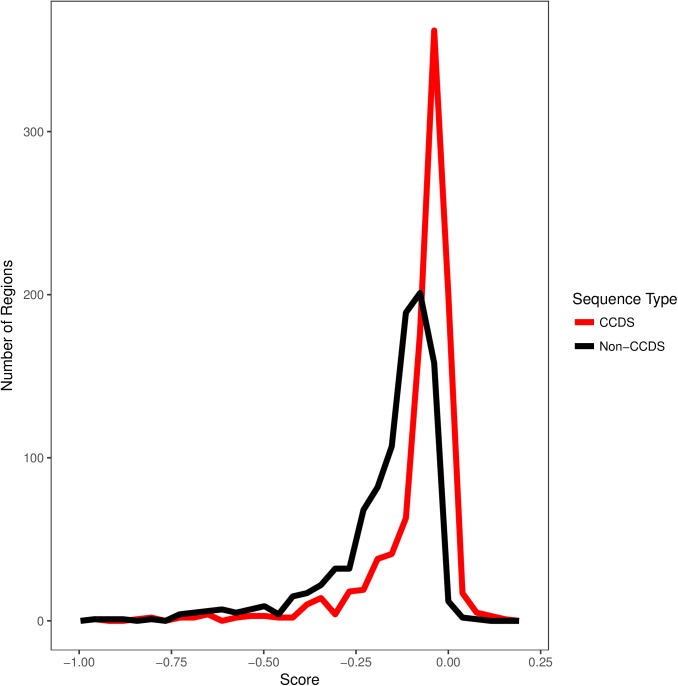
Smoothed histogram of CCDS and non-CCDS Orion scores. For visual clarity scores below -1 were removed, for a total of 996 CCDS scores and 989 non-CCDS scores. The scores were calculated using a control cohort of 1,662 WGS samples. The distributions differ significantly (Permuted Mann-Whitney U test P value: 0.001). The CCDS scores’ variance is 0.023 and the non-CCDS scores’ variance is 0.042.

We found that the mean CCDS Orion score is -0.0874 and the median is -0.0448, while the randomly sampled size-matched non-CCDS mean Orion score is -0.177 and the median is -0.118, and the overall distributions differ significantly (Permuted Mann-Whitney U test *P* value: 0.001). Thus, the CCDS scores are more intolerant overall (Fig 1) indicating that Orion can detect the intolerance of exons as did earlier gene based intolerance scoring methods. As the genes for this test were selected randomly, some of the genes will not be intolerant, and it is therefore not surprising that there is some overlap between the distributions (Fig 1).

**Fig 2:**
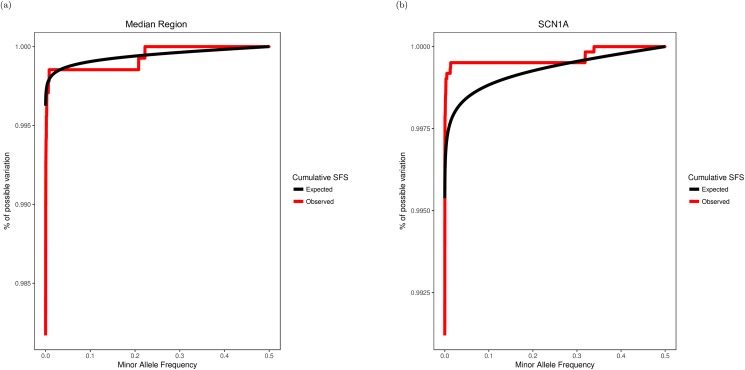
Cumulative SFS visualizations of the median non-CCDS region (a) and SCN1A (b). For both panels, the observed SFS (red) was calculated using a control cohort of 1,662 WGS samples and the expected SFS (black) was calculated using neutral theory.

To visualize the SFS for some of these regions, we plotted the cumulative SFS of the non-CCDS region with the median non-CCDS Orion score (Fig 2A). We also plotted the cumulative SFS of the *SCN1A* gene, which is known to cause disease when mutated[4,8] and scores as highly intolerant using previously introduced gene based intolerance scores such as RVIS[4] (Fig 2B).

Note that the median non-CCDS region does not match expectation (Fig 2A). As stated previously in this article, this is not surprising, as the assumptions used in constructing the neutral model do not hold due to demographic effects such as bottleneck-expansion. In order to assess the intolerance of these regions we need to compare the relative the magnitude of deviation from neutrality.

There is a comparatively clear excess of rare variation in the *SCN1A* cumulative SFS plot (Fig 2B), indicating that variation in this gene rarely becomes common. This matches our expectation that the *SCN1A* protein-coding sequence of the genome is under purifying selection.

We further wanted to compare these scores to the RVIS intolerance scores [4] that we previously developed to assess genic intolerance to functional variation. In the RVIS formulation, a lower score indicates higher intolerance. We found a significant correlation between the Orion CCDS scores and RVIS (Pearson’s r = -0.26, *P* value: 2.9x10^-241^, 95% confidence interval: [-0.27, -0.24], 15,965 genes). We expect the RVIS approach to be more predictive of protein-coding genic intolerance, as it differentiates between synonymous and non-synonymous mutations, and further is based on a much larger cohort (n = 6,503) allowing it to have a much higher resolution for low allele frequencies.

We next assessed the Orion CCDS scores’ relationship with known OMIM disease genes (S2 Data File), using the same methodology we previously used in assessing RVIS [4]. We found the Orion CCDS scores to be predictive of a number of the OMIM disease gene lists (Table 1).

**Table 1:** AUCs and logistic regression P-values for Orion scores compared against OMIM disease gene lists. For each gene list, every OMIM gene was assigned a 0/1 denoting absence/presence in the assessed list.

	AUC	Logistic Regression P-Value	Number of Genes
**Haploinsufficiency**	0.74	1.08x10^-15^	171
**Gain of Function**	0.66	4.73x10^-6^	189
**Essential**	0.70	1.84x10^-87^	2,222
**Seizure Orthologs**	0.66	0.00026	92
**Lethality Orthologs**	0.73	1.9x10^-8^	84
**Non-OMIM**	0.43	2.41x10^-24^	13,858

### Implementing Orion genome-wide

Encouraged by these results, we implemented a sliding window approach to generate genome-wide scores. We use an odd window size so that we can calculate the Orion score of the entire window and assign it to the middle base. We selected a window size of 501bp and applied this approach across all autosomal chromosomes. We excluded bases falling in repeat regions (Methods). The resulting scores are publicly available (https://doi.org/10.6084/m9.figshare.4541632.v1) and can be extracted and downloaded for a given region (www.genomic-orion.org).

We assessed the sliding-window Orion scores' behavior across the *SCN1A* gene. We selected 1000 random Orion scores from *SCN1A*' s introns and exons. We found statistically significant enrichment of higher Orion scores in the exonic regions when compared with the intronic regions (Permuted Mann-Whitney U test *P* value: 0.001). Specifically, exons had a mean and median of -0.174 and -0.171 respectively, while introns had a mean and median of -0.325 and -0.306.

### Comparing Orion scores to key features of the human genome

Following this observation across a single gene, we sought to assess the Orion score genome-wide. For this assessment, we examined whether regions that are known to be intolerant are enriched for higher Orion scores. We tested for enrichment of intolerance in three region types: protein-coding exons; UCNEs; and DHS regions. To attain an empirical-based control (presumed neutral) distribution, we selected 100,000 random Orion scores from across the genome that did not overlap with repeat regions as defined by RepeatMasker [11] (accessed November 2016) or overlapping any of the three region types described above.

Following this, for each regional annotation we randomly selected 100,000 Orion scores and assessed whether there is enrichment for higher Orion scores when compared to the control distribution (Table 2).

**Table 2:** Enrichment of higher Orion scores across regions. We found that exons are clearly enriched for higher Orion scores over the control distribution. This finding is expected, given the selective pressure on the protein-coding region.

	Mean	Median	Permuted MW
**Non-Annotated**	-0.363	-0.336	N/A
**Protein-Coding**	-0.262	-0.241	0.001
**UCNE**	-0.242	-0.226	0.001
**Intersected DHS**	-0.200	-0.175	0.001

Next, we assessed the relationship between the Orion scores and non-coding regions of the genome that are ultra-conserved. We randomly collected 100,000 Orion scores falling in UCNEs, which are defined as non-coding regions greater than 200bp in length that are identical between human and chicken [12]. We found that the Orion scores falling in UCNEs significantly differ from the control distribution (Permuted Mann-Whitney U test *P* value: 0.001). Thus, there is clear enrichment of intolerant Orion scores in these regions. Strikingly, the UCNE scores' mean and median are greater than the exonic scores' mean and median.

Finally, we sought to assess the DHS regions. These regions of open chromatin are enriched for regulatory sequence [13]. For this assessment, we examined the intersection of DHS regions open in all cell types (Methods, S3 Data File). We hypothesized that these regions are likely enriched for regulatory elements associated with genes that are crucial for cell function and would therefore be highly intolerant. We found that these scores are indeed enriched for scores higher than the control distribution (Permuted Mann-Whitney U test *P* value: 0.001), and appear to have the most intolerant score population of the three regions assessed. Furthermore, this finding provides evidence that the Orion scores can indeed capture regulatory regions that are intolerant to variation.

As the Orion approach is solely based on variation in the human population, we sought to assess conservation in a similar framework and compare to our results. We used GERP++[14] as our measure of conservation. We collected the GERP++ scores across the exact same coordinates we used in the Orion evaluations and tested whether the annotated regions were enriched for higher, more conserved, GERP++ scores (S4 Data File).

We found that both exons and UCNEs are enriched for higher GERP++ scores (Table 3). These results were expected, given that the protein-coding genome tends to be well-conserved and UCNEs are defined by conservation.

**Table 3:** Enrichment of higher GERP++ scores across regions.

	Mean	Median	Permuted MW
**Non-Annotated**	-0.175	0.140	N/A
**Protein-Coding**	2.89	4.19	0.001
**UCNE**	5.06	5.5	0.001
**Intersected DHS**	-0.041	0.377	0.001

Strikingly, we found that the GERP++ scores were the lowest in the DHSs compared to the other assessed regions, while the Orion score values for the DHSs were the highest amongst all compared regions. This finding supports previous evidence [15] that these regulatory regions appear to be undergoing human lineage-specific purifying selection. Further, this indicates that the Orion score is well-positioned to detect such purifying selection.

Overall, this set of analyses indicates that known intolerant regions are indeed enriched for higher Orion scores, providing evidence that the genome-wide Orion scores are capturing intolerance.

### Defining the Orion regions

The Orion scores are regional scores, as they are constructed based on a window that includes the surrounding bases. As such, we view the Orion scores not as variant level scores, but rather as measures that can be used in the detection of stretches of sequence that are intolerant. We aimed to detect such stretches of sequence and designate them as Orion regions.

The interpretation of the Orion scores is not in their absolute values, but rather in their value relative to other Orion scores. We therefore sought to detect stretches of sequence that are empirically matched in their characteristics to known highly intolerant prot**e**in-coding exons. We used model-controlled flooding [16] (MCF), a methodology to detect stretches of sequence that fit a particular set of criteria. We set these criteria to match the score population of the most intolerant exons (Methods). Thus, we defined Orion regions as stretches of 100 to 1000 base pairs with a minimum mean Orion score and minimum median Orion score of -0.08, and containing no Orion score less than -0.1.

These parameters can be tuned by the user, depending on the type of regions that need to be detected. The code implementing the MCF is provided on GitHub (https://github.com/igm-team/orion-public).

Using these criteria, we generated a set of Orion regions. We then filtered these regions to remove any overlap with repeat regions (Methods). The Orion regions occupy a total of 4% of the non-repetitive autosomal genome (S5 Data File). Though the regions in this set are empirically matched through their Orion scores to the most intolerant protein-coding exons, 91% of the sequence within the Orion regions does not fall within CCDS. Therefore, these regions denote a portion of the non-coding genome that is highly intolerant.

### Using Orion to prioritize variants observed in patients

We tested whether Orion regions are predictive of known non-coding pathogenic variation. For this analysis, we constructed two sets of variants. The first set consisted of ClinVar [17] (accessed November 2016) “Benign” variants that were annotated as non-coding based on the variant effect predictor [18] (VEP, version 84) (n = 5,031). The second set consisted of ClinVar “Pathogenic” variants that were annotated as non-coding (n = 223).

As the Orion scores are constructed based on human variation, we needed to exclude common variation from the ClinVar benign set of variants. Otherwise, variants that were found to be benign due to being common polymorphisms may be present in the cohort we used to calculate the Orion scores and therefore lead to more tolerant Orion scores. To avoid this potential bias, we removed all ClinVar benign variants with a 1000 Genomes [19] MAF above the minimal MAF possible in our cohort (Methods). We also wanted to avoid potential bias due to regional annotations. We therefore randomly subset the variant sets so that their annotations matched. This left us with 74 variants in each set (S6 Data File). Each final set contained 13 UTR variants, 60 intronic variants, and one variant falling upstream of a protein-coding gene.

Encouragingly, we found that only 3 of the benign variants (4%) fall in Orion regions, while 11 (14%) of the pathogenic variants fall in Orion regions (Fisher’s Exact Test P-value: 0.046, odds ratio: 4.1). This result provided further evidence that we can use the Orion methodology to capture pathogenic mutations.

One of the most important potential applications of Orion is in scoring the mutations observed in patients thought to have a genetic condition. To assess the utility of Orion scores in this context, we evaluated whether *de novo* mutations (DNMs) seen in patients with presumed genetic conditions fall preferentially in Orion regions relative to DNMs found in unaffected controls. As we did not have a large cohort of matched case / control high-confidence non-coding DNMs, we relied on two exome-sequencing cohorts to test this: a cohort of DNMs found in patients with autism [20] and a cohort of DNMs in patients with epileptic encephalopathies [21]. We used the controls provided in the autism study as controls for both cohorts of patients (Methods).

We first tested whether there was enrichment for case DNMs from the autism cohort falling into Orion regions. We did not find evidence of a significant enrichment (Fisher’s Exact Test *P* value: 0.960, Odds ratio: 0.99). We next performed the same test on the epilepsy cohort. We found that within the epilepsy cohort, 60 out of 382 (15%) of the DNMs fell within Orion regions, while 192 out of 1,745 (11%) of control DNMs fell within Orion regions (Fisher’s Exact Test *P* value: 0.014, Odds-ratio: 1.5). This confirms that case *de novo* mutations are preferentially drawn from Orion intolerant regions.

## Discussion

Interpreting variation in the non-coding region of the genome has remained one of the central challenges of human genetics. Currently, whole-exome sequencing (WES) remains the most common application of next-generation sequencing in clinical settings. WES diagnostic studies have indisputably transformed gene discovery in diseases—especially for severe sporadic diseases, in which trio sequencing typically yields a diagnostic rate of around 25% [22–24]. Despite this success, a large fraction of patients remain undiagnosed, pointing to the necessity of investigating non-coding regions of the genome.

The Orion methodology has given us a novel view of the genome and equips us with a new ability to interpret variation in the non-coding human genome. There are both immediate and longer term applications of these scores. We anticipate that researchers will immediately use Orion scores to prioritize the variants observed in patients with presumed genetic conditions and that these scores will dramatically improve the interpretability of whole-genome sequence data in patients that are found not to have pathogenic mutations after exome sequencing [22–24]. Recognizing the importance of the Orion scores in patient settings, we have provided a web interface for viewing the Orion scores across a given set of coordinates (www.genomic-orion.org). It is also worth emphasizing that the resolution of Orion scores will increase dramatically as the size of the sample used to define the scores increases. We further anticipate that regions defined by Orion will immediately be used to implement collapsing analyses analogous to what has been applied to genes, but using Orion regions as the “elements” in such analysis. This will facilitate the development of powerful whole-genome sequence based collapsing analyses, as for example envisioned in the Centers for Common Disease Genomics work [25].

Beyond improving the scope of diagnostic sequencing studies, these scores have opened the floodgates for interrogating the biology of the non-coding genome. We have shown that of the regions we have assessed, the intersection of DHSs open in all cell types is both the most securely intolerant region based on Orion and the least conserved region based on GERP++. These results may indicate that Orion-like approaches, formulated entirely based on human standing variation, will perform much better in identifying pathogenic regulatory mutations than methods based on phylogenetic conservation. An interesting follow-up analysis could examine whether the putative regulatory regions of known dosage-intolerant genes [3] are enriched for higher Orion scores when compared to dosage-tolerant genes.

Many important questions remain about whether DHSs from specific tissues are more intolerant than others, the functional importance of UCNEs, and the distribution of Orion scores for other annotated regions of the genome. Performing in-depth interrogations to answer these questions is a clear next step for Orion.

## Methods

### Calculating the Orion score

The Orion score is defined as the departure of the observed SFS within a cohort of human WGS data from the expected SFS. Let n be the number of samples in the cohort. The observed SFS is defined as the vector H with n + 1 elements:

$$H=(\eta_{0},\eta_{1},\eta_{2},\ldots ,\eta_{n}).$$

Where η_i_ is equal to the number of sites for which i samples have the minor allele.

The expected SFS is calculated based on neutral theory [7] as follows. Let μ be the haploid mutation rate per generation of a given region, based on a tri-mer mutation matrix [8]. Let N_e_ be the effective population size, which we have set to 10,000[7]. Let θ be the expected number of mutations introduced into the population per generation. For diploids:

$$\theta =4*N_{e}*\mu .$$

The expected SFS is defined as the vector Ψ with n + 1 elements:

$$\Psi =(\psi_{0},\psi_{1},\psi_{2},\ldots ,\psi_{n}).$$

Where:

$$\psi_{i}=\theta *\frac{\frac{1}{i}+\frac{1}{n*2-i}}{1+\delta_{i,n*2-i}},\;i=1..n,$$

where δ_i,n*2−i_ = 1 if i = n * 2 − i, otherwise it equals 0.

We extend this to include $\psi_{0}=\kappa -\sum_{i=1}^{n}\psi_{i}$, where κ is the number of bases in the assessed region.

We then calculate the weighted mean point-by-point difference between the two SFSs. The point-by-point difference is calculated by subtracting the expected from the observed, so that a higher score indicates a more intolerant region. These differences were then weighted using a weighting vector constructed based on the corresponding minor allele frequency. Let P be the vector of weights, such that:

$$P=(\rho_{0},\rho_{1},\rho_{2},\ldots ,\rho_{n}).$$

Where:

$$\rho_{i}=\frac{2n}{i+1}.$$

Thus, differences with lower MAFs carry more weight than those with higher MAFs. Following this, the each element in the vector P is divided by the sum of all elements in P.

The final weighted mean is then divided by θ, to account for differences in mutability, and multiplied by 100,000 for convenience. The final resulting value is the Orion score.

### Sample processing

We began with whole-genome sequencing (WGS) samples from two sources: those sequenced internally at the Duke Center for Human Genome Variation/Columbia University's Institute for Genomic Medicine and those sequenced as part of the Simons Simplex Collection (SSC)[26], (n = 729 and 2,076, respectively). The Columbia University Medical Center Institutional Review Board and the Duke University Health System Institutional Review Board approved the study protocol and informed consent was obtained from all subjects.

The sequencing and processing strategies differed slightly based on the data that we had available. Samples sequenced internally employed Illumina's HiSeq 2000 platform to 30x mean coverage and were processed by the IGM's standard WGS pipeline. We used Edico Genome's field-programmable gate array DRAGEN system to perform alignment for performance [27]. Note that in this step we departed from the Genome Analysis Toolkit's (GATK) WGS best practices workflow which relies on BWA [28] for alignment. However, public sources indicate high concordance rates between DRAGEN and standard GATK best practices' [27]. After this step, we follow GATK WGS best practices workflow, using GATK version 3.4 and GATK's HaplotypeCaller and GRCh37. SSC samples were sequenced by the New York Genome Center on an Illumina HiSeq X Ten to 30x mean coverage. SSC samples were subsequently processed according to GATK version 3.4 best practices.

As per a pre-established non-relatedness condition, we excluded all related samples from our cohort. We first removed all children (n = 1,038) from the quads represented in SSC and ran KING[29] on a set of 4,081 well-covered variants of intermediate frequency pruned for linkage disequilibrium in order to find and remove a minimal set of 105 second-degree or greater relatedness samples in order to arrive at a set of samples with no pairwise second-degree relationships (n = 1,662; 624 internal, 1,038 from SSC). Lastly, all samples' genomic Variant Call Format (gVCF) files were joint-genotyped together to test all samples for evidence of variation in conjunction with all other samples' data and perform Variant Quality Score Recalibration (VQSR), in which we set the 99.90–100.00 tranche to failures.

### Sliding-window Orion score calculation

The data that we have to work with for Orion score calculation are in their raw form, a VCF, one or more gVCF files, and a file containing mutation rates by tri-nucleotide context which was generated from data published publicly and normalized[30] to a mean of 1.2x10^-8^. We pre-process this data into simple file formats in order to decouple the complications inherent in these file formats and the score calculations. Our input files are: 1. mutation rates, 2. coverage/capability to confidently genotype, and 3. allele counts by position.

As noted elsewhere, we calculate our expected SFS based on the mutation rate in the window of interest. We use a custom python script to calculate a mutation rate value for all positions in the genome, other than those containing an N or at the beginning/end of a chromosome as the method uses the tri-nucleotide context centered on the base of interest.

The second input file generated is a summary by position for all samples of either read depth or genotype quality (GQ). We chose to summarize GQ as ultimately we wanted to be able to remove bases from consideration if we could not confidently genotype those positions, regardless of whether they had reads aligning. Our custom python scripts summarize, for every genome position, the fraction of samples that have > = 20 GQ based on the gVCF data.

The third input file used summarizes the VCF's variant calls genome-wide. We focus only on the single-nucleotide variants (SNV). For any SNV site not in the lowest quality tranche per VQSR, we retain the site if > = 50% of the potential alleles are genotyped at a GQ > = 20. We then multiply the fraction of covered alleles that are non-reference by the total number of alleles in the sample set (including uncovered) and designate this as the allele count (AC). Lastly we fold the AC if necessary. In other words, if the alternate allele frequency is greater than 50%, AC is updated to equal the total number of alleles in the sample set minus the AC. Therefore, the AC is always less than or equal to half of the total number of alleles in the sample set.

Our Orion score calculations are implemented according to the methods described in 'Calculating the Orion Score' in another python script. Our standard approach is to not calculate a score if < 50% of sites in the window do not have adequate coverage (< 70% of samples having > = 20 GQ at a site) per the gVCFs summary file. Notably this is substantially faster than the pre-processing described. All scripts described have used the paradigm of tab-delimited files gzipped by bgzip and indexed by tabix [31] to achieve random access and utilizing luigi (http://luigi.readthedocs.io/en/stable/index.html) in a map-reduce manner for massive parallelization and automatic checkpointing/resumption from points of failure.

Small gaps (< = 10bp) in the Orion scores were imputed using linear interpolation.

### Removing repeat regions

Repeat regions were downloaded from the UCSC Table Browser [11] (September 2016). Specifically, we downloaded the hg19 track “RepeatMasker” from the “Repeats” group. We removed all scores falling in repeat regions [32]. Note that since we use windows to calculate scores, some scores’ windows may overlap repeat regions.

### DNAse hypersensitive sites

DNAse hypersensitive sites (DHSs) are associated with regulatory regions of the genome. We thus expected that DHSs that were open in all cell types to be intolerant to variation. For this analysis, we used the UCSC ENCODE regulatory DNAse cluster file (V3), which indicates DHS peaks in 125 different cell types[13,33]. In our evaluation we only used DHSs that are open in all 125 cell types.

### Forward-time population simulator

We simulated genomic data using SimuPOP [9], a forward-time population genetics simulator in order to test the correlation of different scoring formulations with applied selective pressures. Using SimuPOP's script to simulate rare variants (srv.py), we ran 100 simulations with differing selective pressures.

We used an infinite sites model and a mutation rate of 1.8x10^-8^. Each mutation was assigned a selection coefficient, s_i_, drawn from a gamma distribution, S. We ran 100 simulations with increasing shape parameter values, starting with 0 and incrementing by .01 with each iteration; the scale parameter remained constant at .32:

$$\begin{array}{c} S=Gamma(k,\theta )\\ k=[0,0.01,0.02,\ldots ,1]\\ \theta =0.32 \end{array}$$

Assuming *A* is the wild type allele and *a* is the mutant allele, the fitness values at locus was assigned as follows:

$$\begin{array}{c} f_{AA}=1\\ f_{Aa}=1-0.5*S_{i}\\ f_{aa}=1-S_{i} \end{array}$$

Then, the default SimuPOP exponential multi-locus selection model was used to assign individual fitness values to each parent. The fitness of each individual (f_individual_) is a function of the fitness values at each locus in that individual's genome. Let *f* be the fitness of the individual and S_i_ be the selection coefficient for allele:

$$f_{individual}=e^{-\sum i*(1-S_{i})}$$

We evolved an initial population of 8,100 individuals with 63,000 base pairs for 81,000 generations and expanded them to 900,000 individuals in 370 generations after a 100 generation bottleneck of 7,900 individuals [34]. These particular rapid population expansion demographic parameters have been used previously to produce site frequency spectra that closely match those observed in a real European population [34,35].

The simulated multi-stage demography acts as an idealized model of recent explosive human population growth that resulted in an excess of rare variation. Thus, we believed that employing this model would allow us to distinguish whether certain scoring frameworks were more sensitive in distinguishing between rare variation that occurred due to selection versus rapid population expansion.

### Defining Orion regions

In order to define the Orion regions, we took the top 5% most intolerant exons[4], based on the ExAC_OEratio score on the genic intolerance website (www.genic-intolerance.org, S7 Data File). We calculated the mean, median and minimum scores for each of the intolerant exons. For each of these measures, we took the mean across all exons and added one standard deviation. These values were set as our minimums in detecting Orion regions.

### Window size selection

We experimented with three window sizes: 251bp, 501bp, 1001bp.

For each window size, we calculated the distribution of the number of unique variants within each window based on chromosome 20. Given the weighting scheme we used in constructing our score, we were particularly interested in the distribution of variants with low MAFs. Therefore, we filtered the variant distribution to contain only variants with a MAF of 1.5% or lower. We found that the 251bp window had an average of 4.5 variants, the 501bp window had an average of 9.8 variants (S1 Fig), and the 1001bp window had an average of 18 variants.

We sought to select the smallest possible window size in order to successfully capture local stretches of intolerance. Based on these data, we opted to use the 501bp scores.

We further calculated the correlation between the scores and found a Pearson’s r correlation of 0.82 between 251bp and 501bp, a Pearson’s r correlation of 0.69 between 251bp and 1001bp, and a Pearson’s r correlation of 0.86 between 501bp and 1001bp. Therefore, all three window sizes appear to be highly correlated.

### Permuted Mann-Whitney U test

*P* values were generated by conducting a permuted Mann-Whitney U test. The Mann-Whitney U test is a nonparametric test that makes no assumptions about the underlying probability distributions of the assessed data. We first compute the actual Mann-Whitney U two-sided *P* value of the observed data. We then randomly permute the labels of the data and compute additional *P* values n_p_ = 1,000 times. The number of times the actual *P* value is larger than or equal to the *P* values in the list of randomly generated *P* values is designated as G. The permutation *P* value is calculated as: (G+1)/(n_p_ +1).

#### Selecting random scores from regions

We selected random scores from regions by first selecting chromosome, then selecting the position, in order to allow roughly equal representation across chromosomes.

### ClinVar variant extraction

We extracted all ClinVar variants that were annotated as either “Pathogenic” or “Benign”. Variants annotated as both were excluded.

To filter for non-coding, we used the variant effect predictor (version 84), with the most damaging annotation output. We then considered any variant annotated as the following as noncoding: "3_prime_UTR_variant", "5_prime_UTR_variant", "downstream_gene_variant", "intergenic_variant", "intron_variant", "mature_miRNA_variant", "nc_transcript_variant", "non_coding_exon_variant", "upstream_gene_variant".

Following this, we excluded variants with a 1000 Genomes MAF above the minimal MAF possible in our cohort. As our cohort size was 1,662, variants with a MAF above or equal to 1/3324 were excluded.

We then filtered to keep only variants falling within the non-repetitive autosomal genome.

### Case / control analysis

We limited the *de novo* mutations data to single-nucleotide variants only, and excluded mutations present as variants in the NHLBI ESP exome variant calls.

All the mutations in the epileptic encephalopathies data from the Epi4K study are Sanger validated.

In the autism data set, we removed mutations called in both siblings. We also only included mutations for which either at least one of the institutes analyzing the data (Cold Spring Harbor Laboratory, Yale School of Medicine, University of Washington) had validated the mutation, or at least one of the institutes labeled the mutation as a ‘strong’ variant call while no other institute labeled the mutation as ‘not called’ or ‘weak’.

We further filtered the DNMs to keep only variants falling within the non-repetitive autosomal genome.

## Supporting information

S1 Text Simulations to investigate scoring formulations. This file contains the results of our assessments of different potential Orion score formulations.
(PDF)

S1 Table Phenotypes of internal WGS cohort. (PDF)

S1 Fig Distribution of unique variants with a MAF < = 1.5% per sliding window for chromosome 20. Windows with less than 50% of their bases covered were excluded, as these windows were not included in the score formulation. As with the score formulation, a base is considered covered if more than 70% of samples have > = 20 GQ at the assessed base.
(PDF)

S1 Data File A BED file that contains the coordinates used for the comparison of CCDS and Non-CCDS Orion scores. This file contains the coordinates of 1000 CCDS genes and 1000 random stretches of non-CCDS sequence matched in size. The additional column “full_gene_score” contains the Orion score for the full region. For the CCDS genes, this means that each exon of the CCDS gene will have the same score, which corresponds to the Orion score across the entire gene. The additional column “annotation” denotes whether the region is CCDS or not. If it is CCDS, the gene is indicated. If it is non-CCDS, the gene that the non-CCDS region is matched in size to is indicated.
(BED)

S2 Data File A tab-delimited file that contains the gene lists. This file contains a row per-gene denoting which of gene lists the gene belongs to. A zero indicates membership, a 1 indicates non-membership.
(TXT)

S3 Data File A BED file that contains the coordinates of DHSs that are open in all cell types. (TXT)

S4 Data File A zipped BED file that contains the coordinates of random scores selected from different regional annotations. The additional column “gerp_score” contains the GERP++ score of the coordinate. The additional column “orion_score” contains the Orion score of the coordinate. The additional column “orion_coverage” contains the fraction of the Orion score’s window that was covered when calculating the score. The additional column “annotation” denotes the regional annotation that the coordinate was drawn from, with the value “random” indicating that it was drawn from the control distribution as described in the manuscript.
(ZIP)

S5 Data File A zipped BED file that contains the coordinates of the Orion regions. (ZIP)

S6 Data File A BED file that contains the coordinates for the ClinVar variants that were used in the manuscript. The additional column “annotation” denotes whether the variant is labelled as pathogenic or benign. The additional column “most_severe_function” denotes the most severe function of the variant as annotated by the variant effect predictor. The additional column “1kg_maf” contains the global 1000 Genomes minor allele frequency of the variant.
(BED)

S7 Data File A BED file that contains the 5% most intolerant exons, based on the ExAC_OEratio score on the genic intolerance website (www.genic-intolerance.org). The additional column “gene_exon” denotes the gene and exon the coordinates correspond to. The additional columns “mean”, “median”, “min” and”max” respectively denote the mean, median, minimum and maximum Orion scores for this range. The additional column “size” denotes the size of the region, while the additional column”covered_size” denotes the size of the region that was covered when calculating the Orion scores.
(TXT)
