# Correction: Orion: Detecting regions of the human non-coding genome that are intolerant to variation using population genetics

**DOI:** 10.1371/journal.pone.0191298

**Published:** 2018-01-11

**Authors:** Ayal B. Gussow, Brett R. Copeland, Ryan S. Dhindsa, Quanli Wang, Slavé Petrovski, William H. Majoros, Andrew S. Allen, David B. Goldstein

The Data Availability Statement for this paper contains incorrect URLs. The correct statement is as follows: The code used in calculating the Orion scores and the Orion regions is provided on GitHub (https://github.com/igm-team/orion-public) under the MIT license. The datasets generated during the study are either included in this article or are available on the figshare.com repository (Orion scores: https://doi.org/10.6084/m9.figshare.4541632.v1; Orion regions: https://doi.org/10.6084/m9.figshare.4536101.v1; Coordinates of defined Orion scores, non-repeat autosomal regions that were covered in our sample: https://doi.org/10.6084/m9.figshare.4536095.v1).

The award numbers in the first sentence of the Funding section are incorrect. The correct sentence is as follows: This work was supported by the National Institute of Mental Health of the National Institutes of Health under Award Number U01MH105670 and by the National Human Genome Research Institute of the National Institutes of Health under the Centers for Common Disease Genomics Award Number UM1HG008901.
