# Supplementary material for: Orion: Detecting regions of the human non-coding genome that are intolerant to variation using population genetics
Source: PLoS One. 2017 Aug 10;12(8):e0181604. doi: 10.1371/journal.pone.0181604 (PMC5552289; doi:10.1371/journal.pone.0181604)
Supplement: S1 Text — This file contains the results of our assessments of different potential Orion score formulations. (PDF) [file pone.0181604.s001.pdf]

Simulations To Investigate Scoring Formulations

We tested the following potential methods to quantify the difference between the observed and expected SFS distributions: the Kolmogorov-Smirnov (KS) test statistic, the Mann Whitney U test statistic, and the weighted mean difference between the distributions. For the weighted version we weighted the scores by the inverse of the minor allele frequencies.

We used a forward-simulation framework to simulate different selection pressures on human populations. We found that the scoring formulation with the strongest correlation to the simulated selection coefficient was the weighted mean difference (Pearson's r=0.97), with both KS and Mann-Whitney U close behind (Mann-Whitney U Pearson's r=0.95, KS Pearson's r=0.95).

In theory, the final score should be divided by θ in order to account for differences of mutability. To assess this, we constructed a test to indicate whether dividing the score by θ would lead to a more stable score for regions in which the selection coefficients are equal but the mutation rate varies.

We assessed this for the weighted mean difference scoring formulation across three selection coefficients: 0.25, 0.5, and 0.75. We found that for all assessed selection coefficients dividing by θ led to the lowest coefficient of variation (CV). For the selection coefficient of 0.25, the raw score CV was 0.54, while dividing by θ decreased the CV to 0.054. Similarly, for the selection coefficient of 0.5, the raw score CV was 0.56, while dividing by θ decreased the CV to 0.036. Finally, for the selection coefficient of 0.5, the raw score CV was 0.54, while dividing by θ decreased the CV to 0.051.

Thus, we elected to use the weighted mean difference between points on the SFS, divided by θ.
