# Supplementary material for: Orion: Detecting regions of the human non-coding genome that are intolerant to variation using population genetics
Source: PLoS One. 2017 Aug 10;12(8):e0181604. doi: 10.1371/journal.pone.0181604 (PMC5552289; doi:10.1371/journal.pone.0181604)
Supplement: S1 Table — (PDF) [file pone.0181604.s002.pdf]

| **Phenotype** | **Frequency** |
|---|---|
| Alzheimer's Disease | 1 |
| Amyotrophic Lateral Sclerosis | 27 |
| Autoimmune Disease | 1 |
| Brain Malformation | 1 |
| Centenarian | 21 |
| Chronic Human Immunodeficiency Virus Infection | 96 |
| Epilepsy | 4 |
| Healthy Control | 195 |
| Hemophilia | 144 |
| Immune Deficiency | 5 |
| Intractable Diarrhea of Infancy | 1 |
| Neurological Disorder | 3 |
| Neuropsychiatric or Neurodevelopmental Disorder | 36 |
| Obsessive Compulsive Disorder | 39 |
| Schizophrenia | 49 |
| Unidentified Congenital Disorder | 1 |
