# Supplementary figures and images for: Orion: Detecting regions of the human non-coding genome that are intolerant to variation using population genetics
Source: PLoS One. 2017 Aug 10;12(8):e0181604. doi: 10.1371/journal.pone.0181604 (PMC5552289; doi:10.1371/journal.pone.0181604)

Distribution of the Number of Variants  
within Sliding Windows

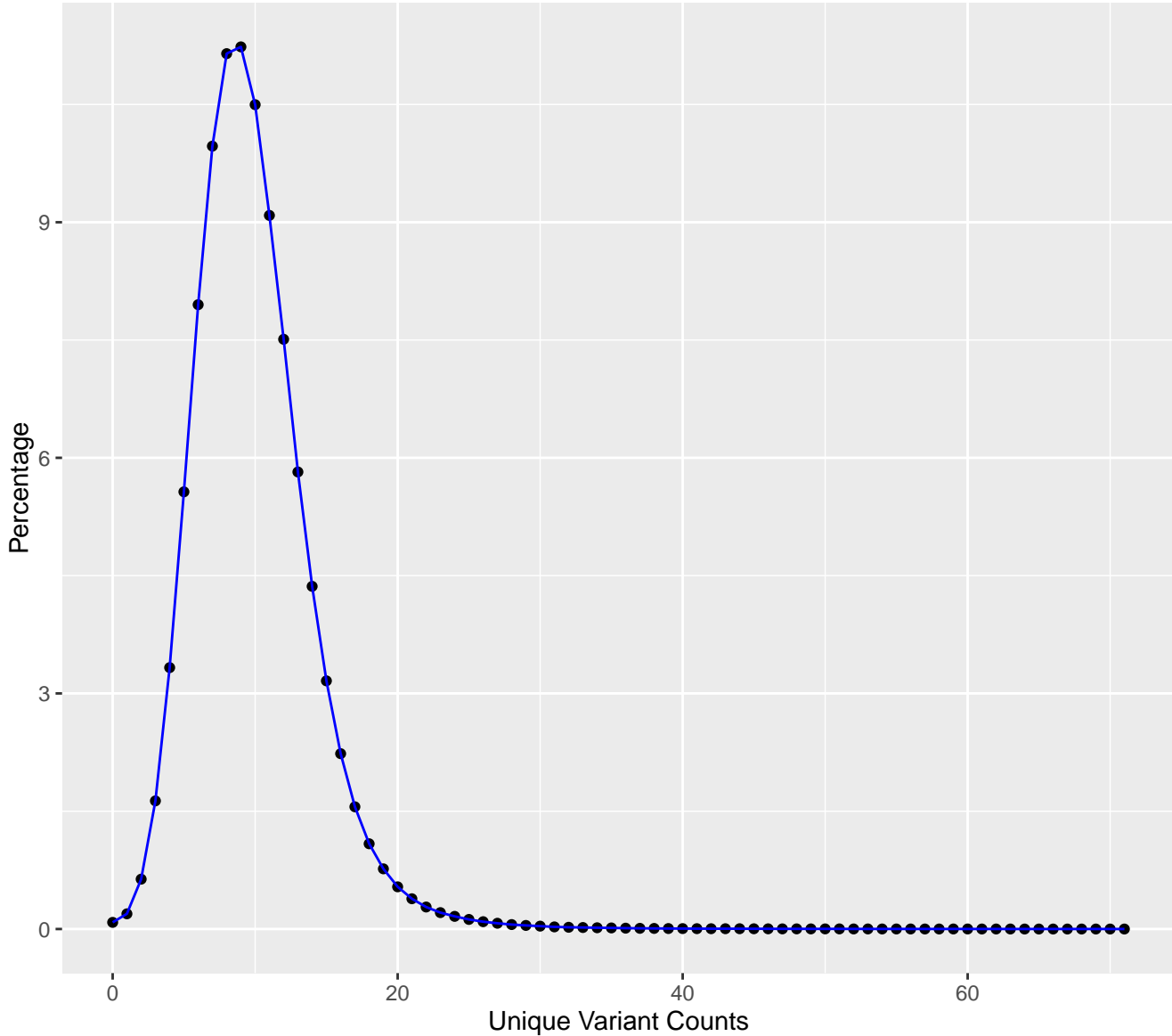

Supplement: S1 Fig — Windows with less than 50% of their bases covered were excluded, as these windows were not included in the score formulation. As with the score formulation, a base is considered covered if more than 70% of samples have > = 20 GQ at the assessed base. (PDF) [file pone.0181604.s003.pdf]
